# Feeding practices, purchasing behaviors, and their association with non-communicable diseases in dogs: Insights from Thai pet owners

**DOI:** 10.14202/vetworld.2025.3174-3186

**Published:** 2025-10-26

**Authors:** Phanuwat Kittitunyapong, Kittikun Kwakkwai, Chanikan Prasertsri, Issariya Sributta, Thita Taecholarn, Kris Angkanaporn

**Affiliations:** 1 Faculty of Veterinary Science, Chulalongkorn University, Bangkok 10330, Thailand; 2 Small Animal Teaching Hospital, Chulalongkorn University, Bangkok 10330, Thailand; 3 Department of Physiology, Faculty of Veterinary Science, Chulalongkorn University, Bangkok 10330, Thailand

**Keywords:** commercial diets, dog nutrition, homemade diets, mixed feeding, non-communicable diseases, pet owner behavior, Thailand

## Abstract

**Background and Aim:**

The rapid expansion of Thailand’s pet industry has influenced dog owners’ purchasing behaviors and feeding practices, with potential implications for canine health. Non-communicable diseases (NCDs) such as obesity, renal disease, cardiovascular disease, and diabetes mellitus are increasingly recognized in companion animals, yet the links between diet and these conditions remain underexplored in emerging pet markets. This study aimed to assess the purchasing and feeding behaviors of Thai dog owners, their knowledge of canine nutrition, and the association between diet type and NCD occurrence.

**Materials and Methods:**

Data were collected between October 2023 and September 2024 using two survey approaches: An online questionnaire completed by 411 dog owners across Thailand and face-to-face interviews with 201 owners at Chulalongkorn University’s Small Animal Teaching Hospital. A total of 612 valid responses were analyzed using descriptive statistics, Chi-square tests, and multiple logistic regression to identify risk factors for NCDs.

**Results:**

Owners prioritized palatability, price, and brand when purchasing food, relying primarily on personal research over veterinary advice. Of respondents, 211 fed commercial diets (valued for convenience and nutritional balance), whereas 190 preferred homemade diets, often lacking supplementation. Mixed feeding was common and carried the highest NCD risk (adjusted odds ratio = 1.783; 95% confidence interval: 1.173–2.710; p = 0.007). Among 169 diagnosed cases, obesity was most prevalent (70.4%), followed by cardiovascular disease (40.2%), renal disease (23.1%), and diabetes mellitus (8.9%). Age was significantly associated with renal and cardiovascular disease, while breed predisposed small dogs to heart disease. Only 35.2% of homemade diets were nutritionally balanced.

**Conclusion:**

Diet type is strongly linked to NCD occurrence, with mixed commercial–homemade feeding posing the greatest risk. Obesity emerged as the most common NCD, with age and breed further influencing disease susceptibility. Knowledge gaps in nutrition and limited veterinary involvement highlight the urgent need for structured educational programs and national guidelines. Within a One Health framework, improving canine feeding practices not only enhances pet welfare but also supports broader household and public health.

## INTRODUCTION

In recent years, the adoption of dogs as companion animals has risen markedly, reflecting a broader societal transformation. A marketing survey [1] reported a substantial year-on-year increase in households owning dogs, a trend closely linked to urbanization, population aging, and the humanization of pets [2]. These shifts highlight the growing perception of pets as family members, with many owners prioritizing canine nutrition even above their own [3]. Despite this increasing concern for pet health, only a small proportion of owners (13%) are knowledgeable and capable of formulating nutritionally adequate homemade diets [4]. In parallel, the Thai pet food market has shown strong growth, reaching USD 1.2 billion in 2021 and projected to expand at a compound annual growth rate of 7.6% between 2022 and 2027 [5]. While research on pet nutrition has largely centered on high-income countries where veterinary input and standardized commercial diets are common [6–8], feeding practices in emerging markets such as Thailand are more strongly shaped by cultural traditions, fresh-market supply chains, and informal advice networks. These contextual factors may alter the risk landscape for diet-related non-communicable diseases (NCDs) in companion animals. Addressing this knowledge gap, the present study investigates how Thai dog owners make feeding decisions and how such practices relate to NCD occurrence, thereby offering region-specific insights with broader relevance to veterinary nutrition strategies across Asia and other emerging markets.

The variety of dog food products available in Thailand has expanded, with manufacturers emphasizing features such as “natural” ingredients to attract consumers [9]. Dog owners also now access nutritional information through a wide range of sources, including social media, advertisements, and online platforms. Nevertheless, the determinants of food purchasing decisions in Thailand remain insufficiently studied, particularly regarding the balance between commercial and homemade feeding practices. This issue is clinically significant, as nutritionally imbalanced homemade diets have been linked to health risks, including bone metabolic disorders, seizures, infectious diseases, and antibiotic resistance [10]. Poor diet quality contributes to the development of NCDs, with obesity representing a leading concern. In the United States, 59% of dogs are overweight or obese, and the prevalence continues to rise [11]. Globally, canine obesity affects 44.4% of dogs in China [12], 42.9% in Thailand [13], 40.5% in Brazil [14], and 21.3% in South Korea [15], predisposing animals to diabetes mellitus, musculoskeletal disorders, and cardiovascular diseases [11]. Other NCDs also present significant challenges, with chronic kidney disease reported in 0.05%–3.74% of dogs in the United Kingdom [16], heart disease in around 10% of those undergoing veterinary examination [17], and diabetes mellitus affecting 23.6/10,000 dogs. Diet plays a pivotal role in both the onset and management of such diseases, partly due to bioactive compounds, including antioxidants and anti-inflammatory agents, that influence metabolic pathways and disease progression [18].

Despite the rapid growth of the pet food industry in Thailand and increasing awareness of canine nutrition, limited scientific evidence exists on how local owners’ purchasing behaviors and feeding practices influence the risk of NCDs in dogs. Much of the current literature on pet nutrition has been generated in high-income regions, where commercial diets dominate, veterinary input is routine, and cultural practices differ significantly from those in emerging markets. In contrast, Thai feeding practices are strongly shaped by cultural traditions, reliance on fresh-market ingredients, and informal advice networks, leading to distinct patterns of homemade and mixed feeding. Existing studies have highlighted that nutritionally imbalanced homemade diets are common and often lack essential supplementation, yet few have quantified their relationship to disease outcomes in companion animals within this regional context. Moreover, while obesity and other NCDs are recognized as major welfare issues globally, data on their prevalence and risk factors among Thai dogs remain fragmented. Critical gaps persist regarding how owners’ knowledge, perceptions, and behaviors interact with diet type to influence disease development. Understanding these relationships is essential for formulating context-specific veterinary guidelines and public health interventions within a One Health framework.

This study was designed to address these gaps by systematically examining the purchasing behaviors and feeding practices of dog owners in Thailand, alongside their knowledge and perceptions of canine nutrition. Specifically, the study aimed to (i) identify the primary factors influencing food purchasing decisions; (ii) characterize owner preferences for commercial, homemade, and mixed diets; (iii) evaluate the nutritional adequacy of feeding practices; and (iv) investigate the association between diet type and the occurrence of major NCDs, including obesity, cardiovascular disease, renal disease, and diabetes mellitus. By combining data from nationwide online surveys and clinical interviews at a veterinary teaching hospital, this research provides robust, region-specific evidence on the links between diet and health outcomes in companion animals. The findings are intended to inform veterinarians, policymakers, and the pet food industry, guiding educational strategies, preventive interventions, and product development to improve canine health and welfare in Thailand and similar emerging markets.

## MATERIALS AND METHODS

### Ethical approval and Informed consent

Ethical exemption was granted as the research involved only surveys of pet owners without experimentation on live animals (IACUC 217/2023). Written informed consent was obtained from all respondents.

### Study period and location

Both online and interview surveys were conducted between October 2023 and September 2024. Two distinct survey types were employed to gather comprehensive data on dog feeding practices. The first was an online questionnaire, designed to investigate factors influencing purchasing behavior and assess owners’ knowledge of feeding practices. This questionnaire comprised two sections: (i) Owner demographic information (e.g., gender, age, and occupation) and (ii) diet-related questions tailored according to the respondent’s choice of commercial or homemade feeding.

The second was an on-site interview survey, conducted with owners of dogs at the Small Animal Teaching Hospital, Chulalongkorn University. This component aimed to examine the relationship between diet type and the occurrence of NCDs, specifically obesity, renal disease, heart disease, and diabetes mellitus. Questions addressed the dog’s demographics, medical history, feeding behaviors, and overall health.

### Study population and sampling methods

Sample size calculations were performed using G*Power version 3.1 (Heinrich Heine University Düsseldorf, Germany). For the online survey, a minimum of 500 respondents was required, based on an effect size of 0.15, a power of 0.80, and an alpha of 0.05. For the interview group, a minimum of 200 respondents was required, based on an effect size of 0.30, a power of 0.80, and an alpha level of 0.05.

### Inclusion criteria

- Online survey: Respondents were eligible if they owned at least one dog, had prior dog ownership experience or feeding responsibility, and were residents of Thailand
- Interview survey: Eligible respondents were dog owners whose pets were hospitalized at the Small Animal Teaching Hospital, were at least 1 year old, and whose owners resided in Thailand.

### Exclusion criteria

- Incomplete responses to the online survey
- Dogs not meeting the minimum age requirement of 1 year
- Owners whose dogs were not treated at the hospital during the interview phase
- Respondents who were not residents of Thailand.

### Data collection tools

A pilot test was conducted with 20 dog owners to refine clarity and resolve ambiguities in the questionnaires. The finalized surveys required approximately 10–15 min to complete, comprising mainly multiple-choice questions with optional open responses.

The online survey was distributed via Google Forms, with each respondent permitted a single submission. The interview survey required owners to answer questions specific to one selected dog undergoing treatment at the teaching hospital. Following participation, owners were provided with educational materials on optimal nutrition and prevention of NCDs in dogs.

### Variables and definitions

#### Diet categories

- Commercial diets referred to packaged pet foods marketed as “complete and balanced” or “complementary” (e.g., treats)
- Homemade diets included meals prepared with human or fresh-market ingredients
- Mixed diets represented combinations of commercial and homemade foods.

#### Disease definitions

- Obesity: Body condition score (BCS) >6/9 or >15%–20% above ideal weight.
- Diabetes mellitus: Persistent hyperglycemia (>120 mg%), glycosuria, and clinical signs [19].
- Renal disease: Functional or structural abnormalities consistent with IRIS stages 1–4 [20].
- Heart disease: Structural or functional abnormalities, most commonly myxomatous mitral valve disease (MMVD) or dilated cardiomyopathy (DCM).

All diagnoses were confirmed by licensed veterinarians.

### Statistical analysis

Descriptive statistics were used to summarize owner demographics and feeding behaviors. The Chi-square test assessed associations between feeding behavior and the prevalence of NCDs. Multiple logistic regression analysis was conducted with NCDs (overall or specific types: Obesity, renal disease, heart disease, and diabetes mellitus) as dependent variables and age group (adult, senior, and geriatric), BCS, neuter status, and diet type (commercial, homemade, and mixed) as independent variables. Cases with missing data were excluded using list-wise deletion.

Data analyses were performed using SigmaPlot 15.0 for Windows (Inpixon GmbH, Germany), with statistical significance set at p < 0.05.

## RESULTS

### Part 1: Online questionnaires

#### Respondent demographics

Out of 548 survey responses, 411 valid questionnaires were included after excluding non-residents of Thailand. The majority of respondents were female (78.1%), aged 18–29 years (54.3%), and undergraduates (32.9%). Household income most commonly fell below Thai Bhat (THB) 10,000/United States dollar (USD) 306 (31.4%) or between THB 10,001 and 20,000/USD 306–612 (27.0%). Dog food expenditure was usually THB <1000/USD 31 (40.6%). Most respondents kept their dogs indoors (70.1%), owned a single dog (50.1%), and did not keep other pets (64.2%).

#### Purchasing drivers and sources of information

**Figure 1:**
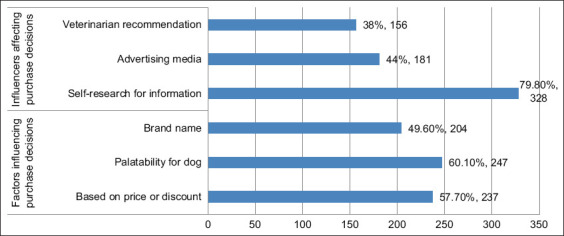
Dog owners’ purchasing behavior based on factors and influencers.

The leading purchasing factors were palatability (60.1%), price or discounts (57.7%), and brand reputation (49.6%). Owners primarily relied on personal research (79.8%), followed by advertising (44.0%) and veterinary advice (38.0%) (Figure 1).

#### Commercial diet practices

Among the 211 owners feeding commercial diets (Table 1), convenience (82.0%), nutritional completeness (76.3%), and availability (56.9%) were the main motivations. Popular brands included Smart Heart (26.5%) and Royal Canin (25.6%). Most owners had been using commercial diets for more than 1 year (67.3%), purchasing mainly from pet shops (65.4%), online platforms (47.9%), and shopping malls (32.2%). Product labels frequently emphasized terms such as “Complete”, “Perfect”, and “Balanced” (78.7%).

**Table 1:** A result of the commercial dog food survey (from 211 responses). The multi-select question was applied in this section.

Question	Answer	Frequency (%)
Reasons for feeding commercial dog food	Owner’s convenience	173 (82.0)
	Nutrition balanced	161 (76.3)
	Accessible	120 (56.9)
Dog food brands commonly fed to dogs	Smart Heart	56 (26.5)
	Royal Canin	54 (25.6)
	Others	52 (24.6)
Duration of feeding	Over 1 year	142 (67.3)
Sources for purchasing	Pet shops	138 (65.4)
	Online retailers	101 (47.9)
	Supermarket/shopping mall	68 (32.2)
Influencing advertising slogans	Complete, perfect, balanced	166 (78.7)
	Hypoallergenic, grain-free, premium	83 (39.3)
	Kidney care, urinary care, low sodium	74 (35.1)
Label information considering	Nutritional information	184 (87.2)
	Ingredients information	174 (82.5)
	Manufacturing date/expiration date	157 (74.4)
Methods of determining food portions	According to the packaging label guideline	119 (56.4)
	Calculating daily energy requirements	82 (38.9)
	*Ad libitum*	72 (34.1)

**Figure 2:**
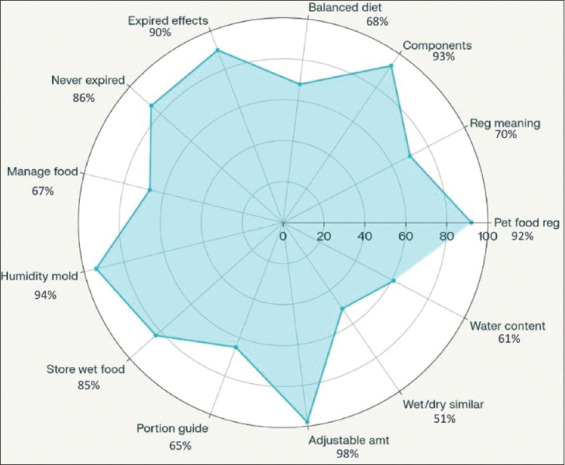
The radar chart presents the percentage of respondents on the evaluation of knowledge and understanding of commercial dog food.

Knowledge assessment revealed high awareness of portion adjustments based on weight and health (98%), risks of damp storage (94%), label interpretation (93%), and product registration numbers (92%). However, only 51% recognized nutritional differences between wet and dry food (Figure 2).

#### Homemade diet practices

Of the 190 owners using homemade diets (Table 2), key motivations were convenience (50.0%), distrust of commercial food (37.4%), and dogs refusing commercial diets (33.7%). Common practices included mixed feeding (52.1%) and rice with meat (38.9%). Portion sizes were usually determined by owner judgment (60.0%), and most had used homemade diets for over 1 year (76.3%). Ingredients were sourced primarily from fresh markets (62.1%) and supermarkets (62.1%), with hygiene cited as the top selection criterion (79.5%). Few owners relied on online recipes (22.1%) or veterinary consultation (14.2%). Vitamin and mineral supplementation was uncommon.

**Table 2:** Responses of a homemade dog food survey (190 responses). The multi-select question was applied in this section.

Question	Answer	Frequency (%)
Reasons for feeding home-cooked food/table scraps	Personal convenience	95 (50.0)
	More reliable than commercial food	71 (37.4)
	Dog refuses to eat commercial food	64 (33.7)
Types of food commonly fed to dogs	Homemade or mixed with commercial food	99 (52.1)
	Rice mixed with meat	74 (38.9)
	BARF	11 (5.8)
Methods of determining food portions	Based on owner’s preference/visual estimation	114 (60.0)
	Using measuring tools	36 (18.9)
	Based on the dog’s preference	33 (17.4)
Duration of feeding	Over 1 year	145 (76.3)
	3–6 months	26 (13.7)
	6 months–1 year	13 (6.8)
Sources of dog food ingredients	Fresh markets	118 (62.1)
	Supermarkets	118 (62.1)
	Convenience stores	61 (32.1)
Criteria for selecting ingredients	Cleanliness and hygiene	151 (79.5)
	Safety standards	119 (62.6)
	Reliable source	103 (54.2)
Sources of BARF recipes	Personal experience	35 (18.4)
	Internet	42 (22.1)
	Consultation with a veterinarian	27 (14.2)
Cleaning/changing dog food bowls	Immediately after feeding	138 (72.6)
	When the bowl looks dirty	32 (16.8)
	3–4 times per week	17 (8.9)
Fat-soluble vitamins and minerals	None given	114 (60.0)
	Calcium	47 (24.7)
	Vitamin C	39 (20.5)
Water-soluble vitamins	None given	143 (75.3)
	Vitamin A	30 (15.8)
	Vitamin D	30 (15.8)

**Figure 3:**
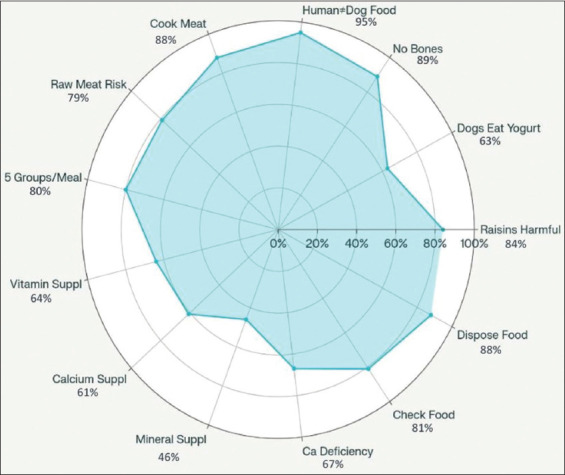
The radar chart presents the percentage of respondents’ knowledge and understanding of homemade dog food.

Knowledge levels varied significantly (Figure 3). Owners showed high awareness in avoiding human food (94.7%), bones (89.0%), and feeding raw meat (87.9%). However, fewer recognized the importance of yogurt (62.6%), vitamin and calcium supplementation (63.7% and 61.1%), or mineral supplementation (45.8%).

#### Use of treats

Among all respondents (n = 411), 83.2% reported feeding treats. The most common treats were dental chews (59.9%), dried meat (53.0%), and stick-shaped products (51.3%). Portion sizes were usually determined by label instructions (57.0%).

### Part 2: Interview surveys

#### Association between diet and NCDs

Diet type was significantly correlated with the occurrence of NCDs (p = 0.024) (Table 3). Dogs on mixed diets had the highest prevalence (62.2%), followed by those on commercial (12.9%) and homemade diets (9.0%). Logistic regression confirmed diet type as a significant predictor (adjusted odds ratio [OR] = 1.783; 95% confidence interval [CI]: 1.173–2.710; p = 0.007) (Table 4). Age group and BCS were not significant predictors.

**Table 3:** The proportion of dogs with NCDs and non-NCDs, categorized by diet type.

Diet	NCDs	non-NCDs
Commercial (Com)	26	10
Homemade (HM)	18	6
Mixed diet (Com+HM)	125	16
Total	169	32

Pearson Chi-square = 7.462 with 2° of freedom (p = 0.024). NCD = Non-communicable diseases (obesity, heart disease, kidney disease, and diabetes mellitus).

#### Prevalence of NCDs

Among 169 NCD cases, obesity was most prevalent (119 cases), followed by cardiovascular disease (68), renal disease (39), and diabetes mellitus (15) (Table 5). While diet type showed a trend toward association with obesity (p = 0.061), age was significantly associated with renal disease (OR = 0.337, p = 0.029) and heart disease (OR = 0.611, p = 0.026) (Table 6). Mixed feeding increased the odds of obesity (OR = 1.480; 95% CI: 1.029–2.128; p = 0.034). Senior dogs (7–11 years) were more prone to obesity, whereas geriatric dogs (≥12 years) were more susceptible to renal and cardiovascular diseases (Table 7).

**Table 4:** Multiple logistic regression of NCD as outcome (one of each four types of NCD; obesity, renal disease, heart disease, and diabetes mellitus) using age group, BCS, and diet type as predictors.

Factor	Adjusted OR	5%–95% CI	Wald statistics	p-value
NCD occurrence	7.523	0.838–68.38	3.212	0.073
Age group	0.635	0.368–1.397	2.655	0.103
BCS	0.350	0.675–1.070	1.916	0.166
Diet type	1.783	1.173–2.710	7.326	0.007

p-values in bold denote significance (p < 0.05). Pearson Chi-square statistic: 200.450 (p = 0.399). Likelihood ratio test statistic: 10.860 (p = 0.013). OR = Odds ratio, CI = Confidence interval, NCDs = Non-communicable diseases, BCS: Body condition score.

**Table 5:** The distribution of NCD cases among dogs based on diet type (commercial diet, homemade, and mix of both diets).

Disease	Commercial diet cases (n = 36)	Homemade diet cases (n = 24)	Mixed diet (n = 141)	Total cases	p-value
Obesity	17	11	91	119	0.061
Renal	7	5	27	39	0.982
Cardiovascular	9	12	47	68	0.131
DM	2	2	11	15	0.887

The total number of cases exceeds the number of dogs in each group because individual dogs may have been diagnosed with multiple conditions (e.g., a dog diagnosed with both obesity and cardiovascular disease). NCDs = Non-communicable diseases, DM = Diabetes mellitus.

**Table 6:** Multiple logistic regression of each NCD as outcome (obesity, renal disease, heart disease, and diabetes mellitus [DM]) using age group, BCS, neuter status, and diet type as predictors.

Factor	Adjusted OR	5%–95% CI	Wald statistics	p-value
Obesity occurrence	1.093	0.126–9.453	0.007	0.935
Age group	0.893	0.576–1.384	0.255	0.614
BCS	0.995	0.830–1.192	0.004	0.953
Neuter status	0.732	0.388–1.383	0.924	0.337
Diet type	1.480	1.029–2.128	1.477	0.034
Renal disease occurrence	0.834	0.019–37.17	0.009	0.925
Age group	0.337	0.127–0.897	4.741	0.029
BCS	0.745	0.487–1.140	1.839	0.175
Diet type	1.475	0.535–4.069	0.563	0.563
Heart disease occurrence	1.970	0.320–12.12	0.535	0.464
Age group	0.611	0.395–0.943	4.944	0.026
BCS	0.917	0.760–1.107	0.808	0.369
Diet type	1.107	0.750–1.633	0.260	0.610
DM occurrence	0.038	0.001–1.263	3.349	0.067
Age group	1.399	0.617–3.171	0.646	0.422
BCS	0.925	0.661–1.293	0.209	0.647
Diet type	1.190	0.577–2.454	0.222	0.638

p-values in bold denote significance (p < 0.05). OR = Odds ratio, CI = Confidence interval, NCDs = Non-communicable diseases, BCS: Body condition score.

**Table 7:** The association between age group of dogs (adult, senior, and geriatric) and each disease of NCDs.

Disease	Adult (n = 30)	Senior (n = 92)	Geriatric (n = 79)	p-value
Obesity	19	63	37	0.014
Renal	2	13	24	0.004
Cardiovascular	4	31	33	0.020
DM	3	8	4	0.565

p-values in bold denote significance (p < 0.05). NCDs = Non-communicable diseases, DM = Diabetes mellitus.

#### Breed associations

Breed type was significantly associated with cardiovascular disease (p < 0.001). Small breeds (<10 kg) were more commonly affected, whereas medium (10–25 kg) and large breeds (>25 kg) showed a tendency toward obesity (p = 0.070) (Table 8).

**Table 8:** The association between breed group (small, medium, and large) and each disease of NCDs.

Disease	Small (n = 126)	Medium (n = 30)	Large (n = 13)	p-value
Obesity	83	26	10	0.070
Renal	28	6	5	0.378
Cardiovascular	63	3	2	<0.001
DM	10	4	1	0.639

p-values in bold denote significance (p < 0.05). NCDs = Non-communicable diseases, DM = Diabetes mellitus.

#### Nutritional balance in homemade diets

**Figure 4:**
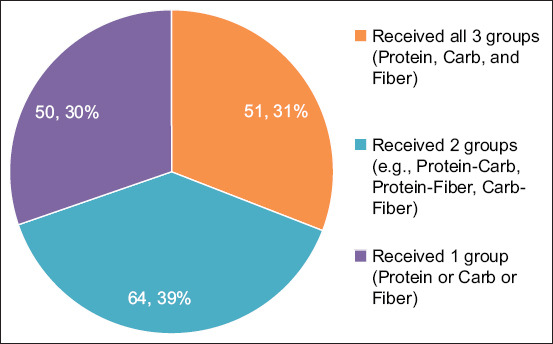
Distribution of frequency and percentage of dogs based on dietary groups (protein, carbohydrate, and fiber).

Among 165 dogs fed homemade diets, only 35.2% received balanced meals including protein, carbohydrates, and fiber. Diets with two components accounted for 44.1%, whereas 20.7% received single-component meals. Chicken was the most common protein source, followed by pork, fish, and liver (Figure 4).

#### Non-dietary health factors

**Figure 5:**
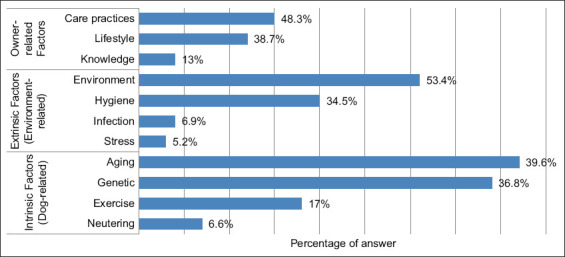
Opinion of dog owners on the other factors influencing dog health.

Owners also recognized additional health determinants beyond diet (Figure 5). Exercise and preventive veterinary care were the most frequently cited, while environmental hygiene and emotional stress received comparatively less attention.

## DISCUSSION

### Overview of feeding practices and knowledge gaps

This study highlights the central role of feeding practices, owner knowledge, and dietary choices in shaping the risk of NCDs in dogs in Thailand. While many owners demonstrated adequate awareness of food safety and general nutrition, several misconceptions and knowledge gaps persisted. These findings reflect the complexity of companion animal nutrition, where inappropriate feeding stems not only from limited knowledge but also from behavioral, cultural, and structural influences.

### Owner knowledge and feeding behavior

Price remained a key factor in food purchasing decisions, with pet shops being the most common source and Royal Canin the most preferred brand [21, 22]. This aligns with earlier reports of Thai owners typically purchasing food from pet shops, spending THB 501–1,000/USD 15.3–30.6 per purchase, 1–2 times per month [23].

Although owners demonstrated strong knowledge of food storage, label interpretation, and safety risks, misconceptions persisted regarding the concept of “complete and balanced diets” and the caloric density of wet versus dry food. Dry dog food typically contains 8%–10% moisture, compared with 60%–87% in wet food [24]. Misunderstanding these differences often led to overfeeding, increasing caloric intake, and increased risk of obesity.

For homemade diets, only 20% of owners included all five food groups in each meal. Most relied on home-prepared meals supplemented with commercial products or rice with meat, with minimal vitamin or mineral supplementation. A study by Hajek *et al*. [25] indicates that 94% of homemade diets lack nutritional adequacy, compared with 57% of commercial diets. Reliance on estimation for portion sizes further increased risks during critical growth phases [26].

Owners generally demonstrated good hygiene practices, including thorough cooking, removal of uneaten food, and regular cleaning of containers. However, raw feeding practices have raised concerns about zoonotic pathogens, such as *Campylobacter* spp. [27]. Reliance on online recipes rather than veterinary consultation mirrored global feeding trends, including in France [28]. This underuse of veterinary expertise is concerning, given veterinarians’ unique ability to tailor diets to physiological needs.

### Mixed feeding and nutritional balance

The strongest epidemiological finding was the association between mixed feeding and the highest prevalence of NCDs. While studies in other contexts suggest that carefully balanced mixed diets may enhance nutrient diversity and gut health [6, 29], Thai feeding practices often resulted in unbalanced nutrition. Only one-third of homemade diets were nutritionally adequate, with minimal supplementation.

The mechanisms underlying increased NCD risk with mixed feeding appear multifactorial:

- Difficulty in portion control, leading to caloric overconsumption [30]
- Dilution of commercial diet quality by nutritionally poor components
- Greater reliance on treats and table scraps.

These patterns parallel human nutrition, where unstructured “diet mixing” contributes to obesity and metabolic disorders [31]. Although therapeutic and human-grade diets may provide benefits by reducing inflammation, supporting the microbiome, and managing weight, further evidence is required [32, 33].

### Obesity and age-related diseases

Obesity was the most prevalent NCD, followed by cardiovascular and renal disease, consistent with global reports that canine obesity is a major welfare concern [34]. Overfeeding, poor nutrient balance, and inadequate monitoring of overall intake, including diets, treats, and scraps, were the primary contributors [35, 36].

BCS and neuter status were not significant predictors, suggesting possible sample size limitations or measurement insensitivity. Age, however, played a major role, with senior and geriatric dogs showing higher risks of obesity, renal disease, and cardiovascular disease. These findings parallel human medicine, where aging populations face increasing burdens of NCDs, underscoring the importance of early preventive strategies [37].

### Breed predispositions

Breed-specific patterns were evident, with small breeds more prone to cardiovascular disease and medium-to-large breeds tending toward obesity. These associations reflect underlying genetic predispositions linked with body size [38]. From a One Health perspective, this mirrors human populations, where genetic vulnerabilities interact with environmental and lifestyle factors. Understanding these predispositions in dogs can, therefore, enhance both preventive veterinary care and comparative disease modeling.

### Treat feeding and cultural practices

Treat feeding was highly prevalent (83.2%), with many owners following label instructions for portion size. While this reflects some adherence to guidelines, treats contributed significantly to hidden caloric intake, reinforcing obesity risk. Similar to human diets, small but frequent calorie additions accumulate over time [34].

Cultural preferences also influenced feeding practices, with many owners opting for homemade diets based on trust in fresh market ingredients. This mirrors Thailand’s human food systems, where fresh foods dominate household nutrition. Such overlaps highlight the need for a One Health approach that addresses shared dietary environments and risk factors.

### One Health and public health implications

The implications of these findings extend beyond veterinary care. Companion animal obesity and chronic disease not only reduce welfare and lifespan but also reflect shared household risks. Clustering of obesity and inactivity between pets and owners has been reported by Westgarth *et al*. [39], suggesting that interventions targeting pet diets and exercise could simultaneously promote healthier lifestyles among owners.

Homemade and raw diets also pose food safety risks, as raw meat can potentially transmit pathogens, such as *Salmonella*, *Listeria*, and *Campylobacter* [40]. This creates household-level hazards, particularly for children and immunocompromised individuals. Veterinarians, therefore, play a critical role in educating owners on nutrient adequacy and safe feeding practices [41].

At a policy level, these results underscore the need for structured nutritional guidelines for pets, parallel to dietary recommendations for humans. Public education campaigns and veterinary-led initiatives could close knowledge gaps, reduce misinformation, and promote evidence-based feeding practices. Embedding pet nutrition within the One Health framework may also enhance public engagement, given the strong emotional and health-related connections between pet and owner well-being [42, 43].

### Limitations

This study faced several limitations inherent to its questionnaire-based and cross-sectional design. The accuracy of the data relied on respondents’ knowledge, honesty, and recall, introducing potential biases such as recall bias and inaccuracies in self-reporting. The absence of longitudinal follow-up restricted the ability to assess temporal relationships or disease progression, including staging and severity, which may influence feeding practices. In addition, the disproportionate number of mixed-diet feeders compared with exclusively commercial or homemade feeders may have biased associations. The final sample size, reduced by exclusion criteria, also limited the national representativeness of the findings. Furthermore, some responses lacked sufficient detail for robust statistical analysis, constraining the depth of interpretation.

### Future implications

Despite these limitations, the study offers valuable insights into dog feeding practices in Thailand, providing benefits for the pet food industry, veterinarians, and pet owners. For the industry, understanding owner preferences and behaviors can inform the development of products that align with both canine health requirements and consumer demand. For veterinarians, the findings highlight critical areas for targeted owner education, particularly regarding the risks of nutritionally imbalanced mixed feeding practices. Such guidance is especially important for new dog owners, where early intervention may help prevent the onset of NCDs.

Future research should extend these findings by addressing unexplored aspects, such as the role of water quality in kidney disease and the specific effects of distinct feeding behaviors on individual NCDs. Controlled dietary trials, nutritional intervention programs (e.g., structured owner education), and longitudinal designs would provide stronger causal evidence. Broader geographic inclusion would also improve representativeness and enhance applicability across diverse contexts. Collectively, such studies would strengthen evidence-based strategies for improving companion animal nutrition and reducing the burden of NCDs.

## CONCLUSION

This study demonstrates that feeding practices and owner knowledge play a pivotal role in the development of NCDs in Thai dogs. Among surveyed owners, mixed feeding of commercial and homemade diets was strongly associated with the highest risk of NCDs (adjusted OR = 1.783), with obesity emerging as the most prevalent condition, followed by cardiovascular and renal diseases. Age and breed also shaped disease susceptibility, with older dogs more prone to renal and cardiovascular disorders and small breeds particularly vulnerable to heart disease. Notably, only one-third of homemade diets were nutritionally balanced, while vitamin and mineral supplementation was uncommon.

The strength of this study lies in its dual-survey design, combining a nationwide online assessment with clinical interviews, which provided both population-level insights and clinical associations. These findings offer the first region-specific evidence from Thailand linking owner purchasing behavior, feeding practices, and canine health outcomes.

From a practical perspective, the results highlight critical areas for intervention. For veterinarians, there is an urgent need to strengthen client education on balanced nutrition, risks of mixed feeding, and appropriate supplementation. For the pet food industry, the data underscore opportunities to develop products that align with both owner preferences and canine health needs. At a policy level, structured nutritional guidelines and public awareness campaigns are warranted to reduce misinformation and prevent diet-related NCDs.

In conclusion, feeding practices in Thailand are influenced by cultural norms, market dynamics, and gaps in owner knowledge, which collectively impact canine health. By addressing these factors through veterinary guidance, industry innovation, and public health strategies, the burden of NCDs in companion animals can be reduced. Such efforts not only improve animal welfare but also resonate with a broader One Health perspective, where healthier pets contribute to healthier households and communities.

## AUTHORS’ CONTRIBUTIONS

KA, PK, KK, CP, IS, and TT: Data collection and curation. PK, KK, CP, IS, and KA: Formal analysis. KA and TT: Methodology and supervision and editing. KA: Project administration. KA, PK, KK, CP, and IS: Drafted the manuscript. All authors have read and approved the final manuscript.
